# Final congenital melanocytic naevi colour is determined by normal skin colour and unaltered by superficial removal techniques: a longitudinal study

**DOI:** 10.1111/bjd.18149

**Published:** 2019-08-07

**Authors:** S. Polubothu, V.A. Kinsler

**Affiliations:** ^1^ Genetics and Genomic Medicine University College London Great Ormond Street Institute of Child Health London WC1N 1EH U.K.; ^2^ Paediatric Dermatology Great Ormond St Hospital for Children NHS Foundation Trust London WC1N 3JH U.K.

## Abstract

**Background:**

Spontaneous lightening of congenital melanocytic naevi (CMN) has not been studied systematically. Final colour is considered an important outcome after superficial removal techniques such as curettage, dermabrasion or laser ablation, and is often compared with colour at birth.

**Objectives:**

To quantify the natural history of CMN lightening over time, and explore phenotypic and genotypic predictors of colour change.

**Methods:**

A longitudinal cohort study was undertaken of 110 patients with CMN (mean follow‐up 5·3 years). Accurate colour‐space measurements were taken from professional serial photographs of CMN and normal skin. Changes in colour over time were modelled using multiple logistic regression, against phenotypic and genotypic variables.

**Results:**

Lightening of CMN was significantly associated with lighter normal skin colour (*P* < 0·001) and with *MC1R* variant alleles (red/blonde hair gene) (*P* < 0·001), but not with CMN colour in the first 3 months of life, *NRAS* genotype or projected adult size of CMN. Importantly, the final colours of adjacent treated and untreated areas of CMN were indistinguishable.

**Conclusions:**

Final CMN colour in childhood is related to the genetically determined skin colour of the individual, is unrelated to the colour of CMN at birth, and is unaffected by superficial removal.

**What's already known about this topic?**

- Final colour of congenital melanocytic naevi (CMN) is considered an important outcome after superficial removal techniques such as curettage, dermabrasion or laser ablation, and is often compared with colour at birth.
- The phenomenon of spontaneous lightening in CMN, in which naevi lighten gradually and sometimes dramatically during childhood, has been described but not systematically studied.

**What does this study add?**

- Final CMN colour in childhood is significantly associated with the individual's normal skin colour, and with *MC1R* genotype, and is therefore genetically determined.
- Final CMN colour is not predictable from CMN colour in the first 3 months of life.
- Superficial removal techniques do not alter the final colour of CMN.

## Abstract 2

**Linked Comment:** https://doi.org/10.1111/bjd.18706.

Congenital melanocytic naevi (CMN) are usually darkly pigmented at birth. Where lesions are multiple or extensive, excision or superficial removal techniques are frequently employed for cosmetic reasons. Final colour of the naevus is considered by clinicians to be an important cosmetic outcome in the context of superficial removal, with lighter CMN deemed to be less noticeable and therefore more desirable, and a lighter final colour deemed an improvement.^1^, ^2^ However, owing to the long‐held and as‐yet‐unsubstantiated belief that early surgical intervention in CMN has better cosmetic outcomes, the decisions to excise or to perform superficial removal are often based on the appearance of the CMN in the immediate postnatal period,^3^, ^4^, ^5^, ^6^ and results assessed by comparison with photographs from that time.

Highly relevant in this context is the known phenomenon of spontaneous lightening in CMN, in which naevi lighten gradually, sometimes dramatically, during childhood.^7^, ^8^, ^9^, ^10^ This is particularly common in scalp naevi,^11^ the reason for which is unknown; however, it has been described at many different sites. This phenomenon could have major implications for cosmetic surgery decisions taken at or near birth. Spontaneous lightening should be distinguished from the very rare cases of actual regression of the naevus.^11^, ^12^, ^13^, ^14^, ^15^, ^16^, ^17^, ^18^, ^19^, ^20^, ^21^, ^22^ It should also be noted that a small percentage of CMN are tardive, defined as appearing fully or partially after birth. These are the only naevi in our experience that appear to darken after birth, although they are, in fact, only developing their full phenotype.^7^, ^8^

What is known about the natural history of pigmentation is that the immediate postnatal period is not representative of the individual's genetic pigmentary phenotype. For example, a baby's hair colour, eye colour and even skin colour will alter in the first year of life, often dramatically. This is thought to be at least partly due to the effects of maternal hormones on pigmentation in the baby, and, after gradual withdrawal of these, the child's genetically determined pigmentation becomes established. In addition, further evolution of pigmentation occurs throughout childhood, with blonde hair, for example, often darkening over time.

We hypothesized that the colour of CMN in the postnatal period may not be related to its final colour, and that final colour may instead be genetically determined. We sought to devise a method to measure lightening in CMN accurately and objectively, to establish the natural colour history of CMN in a large cohort of children, and to look for genotypic and phenotypic associations of lightening.

## Patients and methods

### Patient cohort and colour‐space measurements

**Figure 1:**
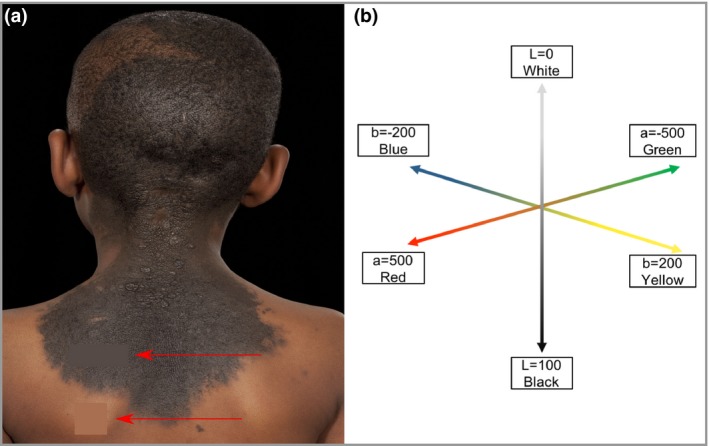
Method of measurement of L*a*b colour‐space values from standardized serial photographs. (a) For each patient, the same representative areas of congenital melanocytic naevi (CMN) and normal background skin were measured over time, with averaging of colour over measurement areas (Adobe Photoshop Elements; Adobe, San Jose, CA, U.S.A.) to reduce the effect of any natural colour heterogeneity in the area. Red arrows indicate areas of ‘colour‐averaged’ CMN and normal skin. (b) Representation of L*a*b* measurements along the three‐dimensional colour‐space axes, where L* is a mathematical value along the black/white spectrum, a* along red and green, and b* along blue and yellow. L* (lucency, lightness) was found to vary the most over time in CMN.

A method of standardized accurate measurement of lightening of CMN over time was devised and optimized, based on published measurements of accuracy of the system chosen.^23^, ^24^, ^25^, ^26^ Serial photographs taken in a professional hospital setting with controlled and standardized lighting conditions (no external light) underwent measurement, and the data were analysed in a blinded manner. For each patient, the same areas of CMN and normal background skin were measured over time, with averaging of colour over measurement areas (Adobe Photoshop Elements; Adobe, San Jose, CA, U.S.A.) to reduce the effect of any natural colour heterogeneity (Fig. 1a). Using the the L*a*b* colour‐space model (CIE‐LAB), mathematical descriptions of all perceivable colours in three dimensions were taken using Digital Colour Meter (version 5·11; Apple, Cupertino, CA, U.S.A.). Briefly *‘*L*’ stands for lucency, representing lightness, a measure of the spectrum between black and white, where L* = 0 is pure black and L* = 100 is pure white. Simultaneously, ‘a*’ is a measure of the spectrum between red and green, and ‘b*’ between blue and yellow (Fig. 1b).

In total, 110 patients for the longitudinal cohort were identified from our tertiary referral paediatric dermatology service. Inclusion criteria were children with a diagnosis of CMN, where *NRAS*,* BRAF* or *MC1R* genotype was available, and where at least one set of professional in‐house hospital photographs was available. Extensive data on phenotype were collected using two separate classifications, the most recently proposed classification from 2013,^27^ and an in‐house classification used over the past 25 years and previously published.^28^, ^29^, ^30^, ^31^ Final colour was defined as the latest measurement available for each patient. Immediate postnatal colour was defined as the first colour measurement within the first 3 months of life where this was available.

### Genotyping

Genotyping data were available on CMN tissue from concurrent studies.^32^, ^33^ Germline *MC1R* genotyping was undertaken on leucocyte DNA using Sanger sequencing (primer sequences as reported previously).^34^

### Statistical analysis

CMN mean colour‐space values were first analysed by anova to identify that L* (lucency) was the most significantly altered of the three colour‐space variables over time. Final L* was then modelled using multiple logistic regression, with the independent variables of sex, age at final photograph, self‐reported ethnicity, L* in the first 3 months of life, final L* of the normal skin, the projected adult size of the largest CMN (as the most widely reported phenotypic variable), *NRAS* mutation positivity and at least one *MC1R* variant allele positivity (SPSS version 24·0; IBM, Armonk, NY, U.S.A.). Bonferroni correction for multiple testing reduced the 95% significance level to a *P*‐value of < 0·006.

### Research ethics approval

This study was approved by the London Bloomsbury Research Ethics Committee.

## Results

In total, 110 patients with CMN were included in the longitudinal cohort. Detailed phenotypic and demographic characteristics are presented in Table 1. Briefly, 38·2% (*n* = 42) of participants were male and 79·4% (*n* = 85/107) had multiple CMN. Mean and median follow‐up was 5·3 and 4·2 years, respectively (range 0·2–16·0). Deep phenotyping was undertaken in all, *MC1R* genotyping in 57·3% (*n* = 63) and *NRAS* genotype was available from affected tissue in 88·2% (*n* = 97) of participants. The total number of photos analysed was 266, with a mean number per patient of three (median two, range 1–7). Mean and median age at the first photograph were 3·1 and 1·3 years, respectively, and at last photograph were 6·8 and 5·8 years, respectively.

**Table 1:** Demographic, phenotypic and genotypic characterization of the longitudinal cohort of patients with congenital melanocytic naevi (CMN)

Sex	
Male	42 (38·2)
Female	68 (61·8)
Self‐reported ethnicity	
White British	67 (60·9)
White other	9 (8·2)
White and black Caribbean	2 (1·8)
White and Asian	1 (0·9)
Indian	3 (2·7)
Pakistani	2 (1·8)
Bangladeshi	3 (2·7)
Any other Asian	2 (1·8)
Black Caribbean	1 (0·9)
Black African	2 (1·8)
Any other black	4 (3·6)
Chinese	1 (0·9)
Not specified	11
Multiple CMN (*n* = 107)	
Multiple	85 (79·4)
Single	22 (20·6)
Missing	3
Projected adult size (cm)	
< 10	27 (24·5)
10–20	20 (18·2)
20–40	16 (14·5)
40–60	13 (11·8)
> 60	32 (29·1)
Multiple medium	2 (1·8)
Satellite numbers at enrolment	
0	22 (21·4)
< 10	18 (17·5)
10–20	11 (10·7)
20–50	10 (9·7)
50–100	15 (14·6)
100–200	16 (15·5)
> 200	11 (10·7)
Missing	7
*NRAS* genotype	
Codon 61 mutation	71 (73·2)
Mutation‐negative	26 (26·8)
Missing	13
*BRAF* genotype	
*BRAF* V600E	4 (4·8)
Mutation‐negative	80 (95·2)
Missing	26
*MC1R* genotype	
At least one *MC1R* variant	39 (61·9)
No *MC1R* variants	24 (38·1)
Missing	47
Neurological and/or developmental problems	
Yes	22 (21·4)
No	81 (78·6)
Missing	7
Magnetic resonance imaging	
Normal	56 (73·7)
Abnormal	20 (26·3)
Missing	34

**Table 2:** Statistics relating to age corresponding to each photo set

	Age at first photo (years) (*n* = 102)	Age at second photo (years) (*n* = 73)	Age at third photo (years) (*n* = 44)	Age at fourth photo (years) (*n* = 25)	Age at fifth photo (years) (*n* = 11)	Age at sixth photo (years) (*n* = 3)	Age at seventh photo (years) (*n* = 3)
Mean	3·2	4·8	6·2	6·8	8·3	6·3	9·1
Median	1·3	3·3	4·9	5·8	8·1	6·7	9·5
Minimum	0·0	0·2	0·4	1·1	3·5	4·9	7·8
Maximum	16·0	14·3	17·4	15·0	16·5	7·1	10·1

**Figure 2:**
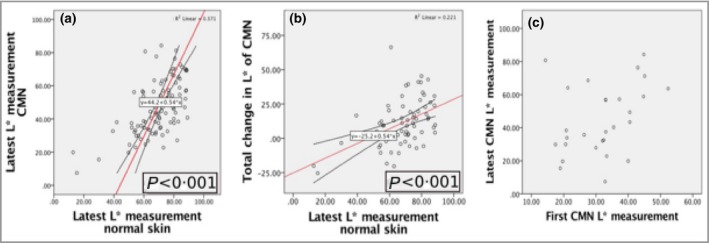
Final congenital melanocytic naevi (CMN) colour and total change in CMN colour are related to normal skin colour, not to colour at birth. (a) Final CMN L* (lucency, lightness) is significantly associated with normal skin L* (*P* < 0·001); (b) total change in CMN L* (or total lightening) of CMN over time is significantly associated with final normal skin L* (*P* < 0·001); (c) no association was demonstrated between CMN L* in the first 3 months of life and final CMN L*.

**Figure 3:**
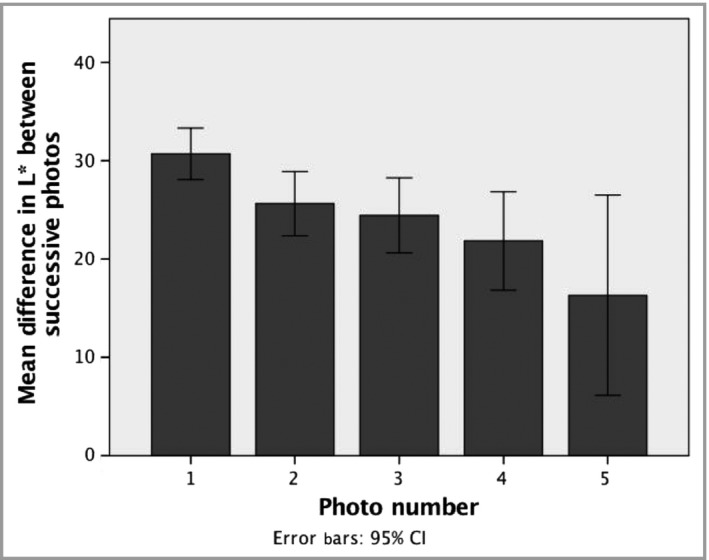
The degree of spontaneous lightening decreases gradually with age. Differences in congenital melanocytic naevi L* (lucency, lightness) shows a decreasing trend with increasing photograph set, which is related to increasing mean age. Photograph sets 6 and 7 had too few data points to be included in this analysis. Error bars represent 95% confidence intervals (CI). Ages corresponding to photo sets are given in Table 2.

Multiple regression analysis modelling final measurement of CMN L* (lucency, lightness) identified a significant positive association with the final measurement of normal skin L* (*P* < 0·001) (Fig. 2a). Modelling of total change in CMN L* over time – in effect total lightening over time – was also significantly and positively associated with the final measurement of normal skin L* (*P* < 0·001) (Fig. 2b). The genetic correlate of these two key findings comes from modelling final CMN L* and total change in CMN L* over time against *MC1R* genotype, where both variables were found to be strongly significantly associated with the presence of at least one germline *MC1R* variant allele (*P* < 0·001). However, when *MC1R* variant status was modelled in association with self‐declared ethnicity, ethnicity was the more powerful predictor and *MC1R* ceased to be significant; those identifying as white had a significantly lighter final CMN colour (*P* < 0·001). The degree of lightening between successive sets of photographs showed a decreasing trend (Fig. 3), implying that the degree of spontaneous lightening decreases with age, although still measurable at photo set 5, at which the mean and median age was 8 years.

No significant association was observed between CMN final L* and the immediate postnatal L* (*P* = 0·064, Bonferroni‐corrected significance level *P* < 0·007) (Fig. 2c). Similarly, *NRAS* genotype and projected adult size of CMN were not found to be associated with CMN final L* or total change in L*. Although age was found to be associated with final CMN L*, this statistical association was negated when combined with normal skin L*. Patient sex was not found to be associated with final L*.

**Figure 4:**
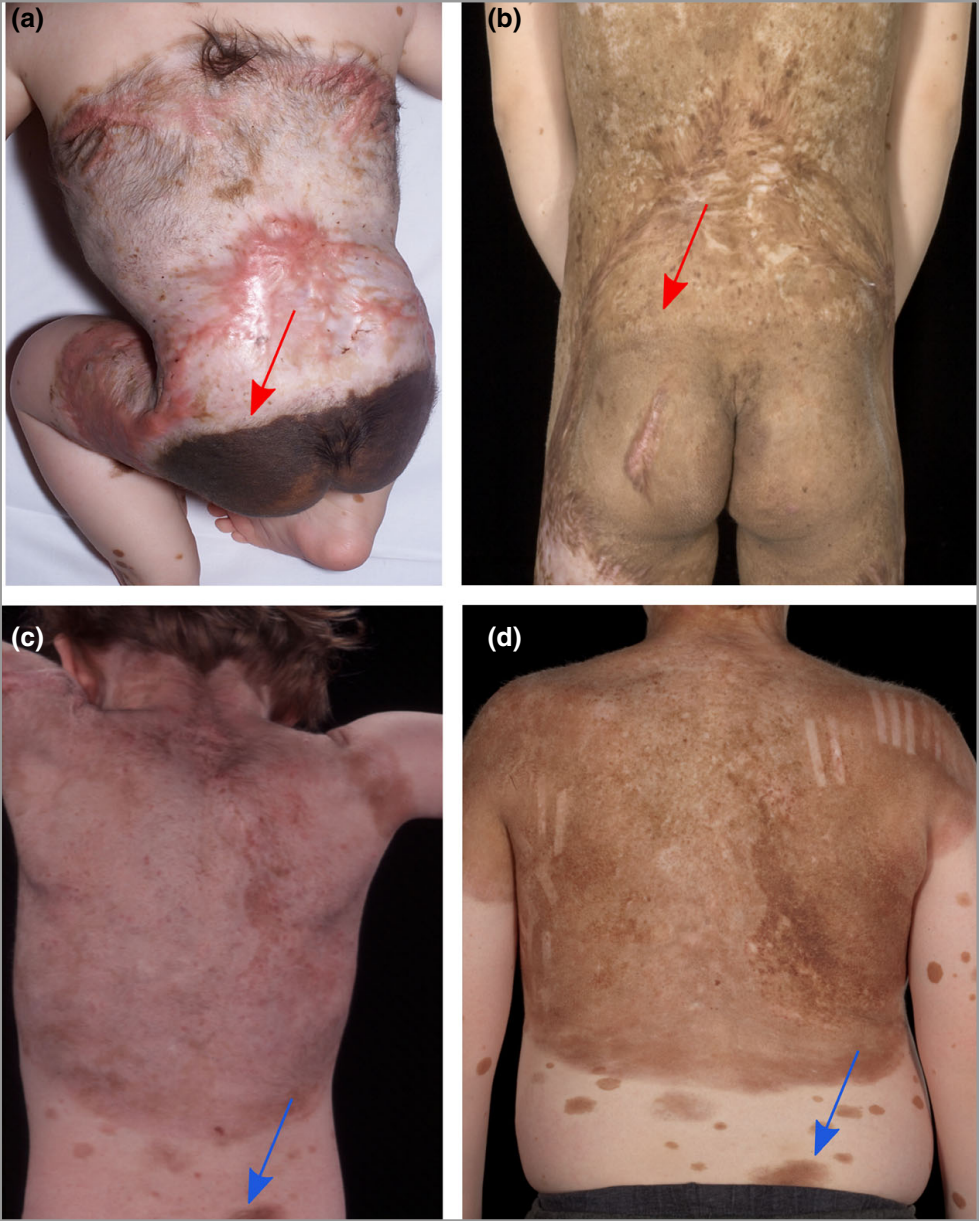
Final congenital melanocytic naevus (CMN) colour is entirely unaffected by superficial removal techniques. Patients immediately postintervention compared with final CMN colour, following intervention with curettage at (a) 9 months and (b) 7 years, and ablative laser at (c) 8 years and (d) 13 years. In (a) and (b) red arrows areas indicate the junction of treated and untreated CMN, and in (c) and (d) blue arrows indicate untreated areas, indicating that the final colour of treated and untreated naevi is the same. Note the scarring from the curettage in (a).

Strikingly, comparison of final colour measurement values from treated and untreated areas of CMN in the same patient, where certain areas had been left untreated for reasons such as site (e.g. the buttocks; Fig. 4a), demonstrated no measurable difference. This finding was observed in a variety of treatment modalities, including dermabrasion, curettage and ablative laser (Fig. 4). When analysis of colour change over time was done, the untreated area had lightened spontaneously, and the treated area had repigmented, both to the same final colour (Fig. 4).

## Discussion

Spontaneous lightening in CMN is a recognized phenomenon but has not previously been studied systematically; as such, clinicians were unable to speculate on the chances of lightening on an individual basis when meeting a child at birth. Decisions on excision or superficial removal techniques are frequently based on the colour of the naevus at, or soon after, birth, and results of superficial removal are often compared with photos from that time, which does not account for any effects of spontaneous lightening. Accurate and objective data on the natural history of colour change in CMN are therefore much needed in the management of patients with CMN.

**Figure 5:**
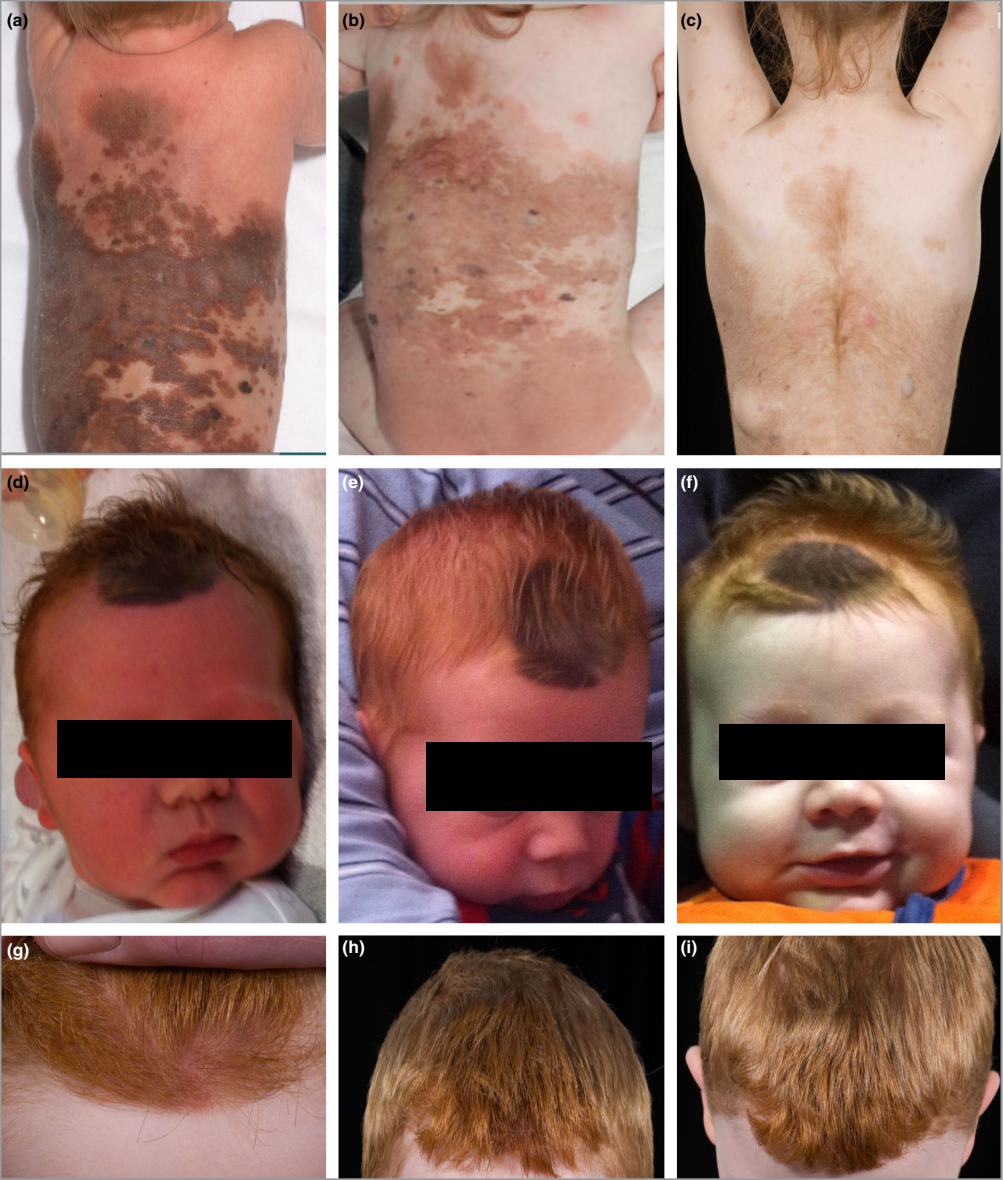
Final congenital melanocytic naevus (CMN) colour is strongly associated with background pigmentary phenotype. A female patient with a bathing trunk CMN at (a) birth, (b) 3 years and (c) 11 years, and a male infant with a frontal scalp CMN at (d) birth, (e) 2 months and (f) 1 year, and later at (g–i) 10 years, demonstrating that the change in CMN colour was strongly correlated to the child's pigmentary phenotype, which is most noticeable in the hair colour.

We find here that final colour of CMN in childhood is genetically determined, related to the background normal skin colour of the individual, and to *MC1R* genotype, which has a strong genetic influence on the lightness of skin colour. This makes a degree of intuitive sense – melanin within naevus cells is assembled from the same mix of melanin monomers as in the rest of the body, albeit in an area of high concentration of naevus cells, and this mix of melanin monomers is known to be genetically determined. This simple rule was beautifully demonstrated in patients in whom hair colour changed markedly over the time of the study, and where the CMN colour could be seen to adopt the same pigmentary characteristics as those of the mid‐brown, then blonde, then red hair in the same individual (Fig. 5). In addition, CMN final colour was unrelated to colour in the first 3 months of life, mirroring what we know of the lack of connection between hair, eye and skin colour at birth, and eventual pigmentary phenotype in childhood. The colour of CMN in the first 3 months of life should therefore not be used as a predictor of final colour, and should not be used as a comparator with which to judge the outcomes of superficial removal at a later date. Instead, parents can be advised that the colour will gradually settle over the first few years, and that more lightening will be expected in lighter‐skinned individuals than in darker‐skinned individuals.

A recent systematic review of laser treatment in CMN concluded that there were good results for reduction of pigmentation in the short term.^1^ However, these results were assessed by eye rather than measurement, by nonblinded physicians, and follow‐up was short, ranging from immediately postprocedure to 2·5 years. Interestingly, despite the positive conclusions, the stated repigmentation rate was 54%.^1^ Furthermore, medical complication rates were high, with reported scarring in up to 25%, and wound infection in up to 18%.^1^ Several small studies have reported dramatic improvements and high levels of patient satisfaction after superficial removal techniques; however, improvements in colour were judged subjectively and immediately postintervention.^5^, ^35^, ^36^, ^37^, ^38^, ^39^, ^40^, ^41^ Furthermore, several reports and studies still advocate early aggressive intervention in CMN with superficial removal techniques with outcomes reported by direct comparison of results to early postnatal pictures.^3^, ^5^, ^38^, ^39^, ^40^, ^41^, ^42^ The striking images here of children in which untreated and treated areas of CMN end up at exactly the same final colour, should bring a halt to superficial removal of CMN as a method for lightening colour.

Interestingly, the phenomenon we describe here may have been the origin of the long‐held theory that early superficial removal was more effective than the same intervention at a later age. If final colour was always compared with birth colour by the physicians and surgeons undertaking these procedures, there would, indeed, have appeared to be more lightening in those who had earlier intervention, particularly in white populations where a large degree of lightening can occur after birth.

In conclusion, final CMN colour in childhood is genetically determined, related to normal skin colour and inherited pigmentary phenotype. In addition, final CMN colour is not related to colour in the first 3 months of life, a time during which hair and eye colour are also under different influences, such as maternal hormones. Therefore, decisions on intervention should not be based upon colour at birth. Children with lighter skin tone will have more lightening of the CMN after birth than those with darker skin tone. Importantly, as final colour is genetically determined it is not affected by superficial removal techniques, after which repigmentation occurs to produce the same colour as the naevus would have naturally taken on over time.
